# (*E*)-3-Methyl-4-[(2-oxidoquinolin-1-ium-3-yl)methyl­eneamino]-1*H*-1,2,4-triazole-5(4*H*)-thione *N*,*N*-dimethyl­formamide solvate

**DOI:** 10.1107/S1600536809050090

**Published:** 2009-11-28

**Authors:** Jia Hao Goh, Hoong-Kun Fun, Adithya Adhikari, B. Kalluraya

**Affiliations:** a X-ray Crystallography Unit, School of Physics, Universiti Sains Malaysia, 11800 USM, Penang, Malaysia; b Department of Studies in Chemistry, Mangalore University, Mangalagangotri, Mangalore 574 199, India

## Abstract

The title 1,2,4-triazole compound, C_13_H_11_N_5_OS·C_3_H_7_NO, crystallizes as a 1:1 dimethyl­formamide (DMF) solvate. The main mol­ecule exists in a *trans* configuration with respect to the acyclic C=N bond. An intra­molecular C—H⋯S hydrogen bond generates an *S*(6) ring motif. In the synthesis, a proton is transferred from the O atom of a hydr­oxy group to the quinoline group N atom. The essentially planar triazole ring and quinoline ring system [maximum deviations of 0.001 (2) and 0.013 (2) Å, respectively] form a dihedral angle of 5.86 (9)°. In the crystal structure, mol­ecules of (*E*)-4-[(2-hydroxy-3-­quinolyl)methyl­eneamino]-3-methyl-1*H*-1,2,4-triazole-5(4*H*)-thione are linked into *R*_2_^2^(8) centrosymmteric dimers *via* N—H⋯O hydrogen bonds. These dimers are further linked into an extended three-dimensional structure by the DMF solvent mol­ecules *via* inter­molecular N—H⋯O and C—H⋯O hydrogen bonds. The crystal structure is consolidated by two different inter­molecular π–π inter­actions [centroid–centroid distances = 3.6593 (12) and 3.6892 (12) Å].

## Related literature

For general background to and applications of 1,2,4-triazole derivatives, see: Al-Soud *et al.* (2003 ▶); Almasirad *et al.* (2004 ▶); Amir & Shikha (2004 ▶); Holla *et al.* (2003 ▶); Turan-Zitouni *et al.* (2005 ▶); Walczak *et al.* (2004 ▶). For the pharmacological properties of quinoline derivatives, see: Janardhana *et al.* (2008 ▶); Kalluraya & Sreenivasa (1998 ▶). For general applications of Schiff base derivatives of 1,2,4-triazole-5-ones, see: Demirbas *et al.* (2004 ▶); Sujith *et al.* (2009 ▶). For hydrogen-bond motifs, see: Bernstein *et al.* (1995 ▶). For closely related structures, see: Dufresne *et al.* 2008 ▶; Fun *et al.* (2009 ▶); Song *et al.* (2008 ▶). For the stability of the temperature controller used for the data collection, see: Cosier & Glazer (1986 ▶).
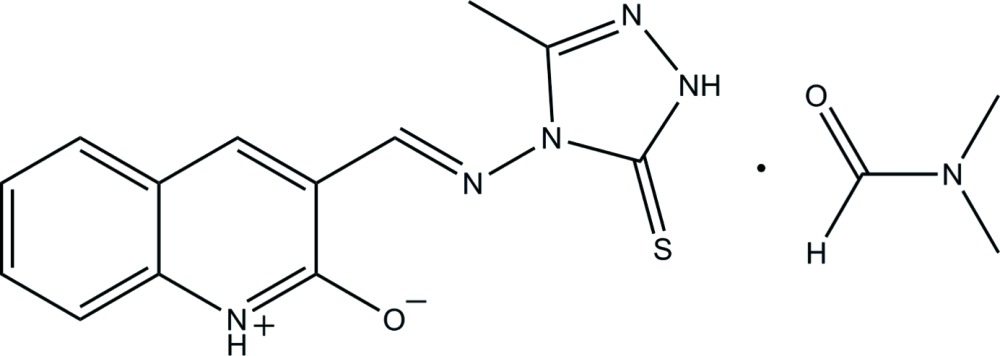


## Experimental

### 

#### Crystal data

C_13_H_11_N_5_OS·C_3_H_7_NO
*M* *_r_* = 358.42
Monoclinic, 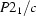

*a* = 7.2374 (1) Å
*b* = 23.4970 (4) Å
*c* = 10.8214 (2) Å
β = 107.820 (1)°
*V* = 1751.97 (5) Å^3^
*Z* = 4
Mo *K*α radiation
μ = 0.21 mm^−1^
*T* = 296 K
0.45 × 0.27 × 0.19 mm

#### Data collection

Bruker SMART APEXII CCD area-detector diffractometer
Absorption correction: multi-scan (**SADABS**; Bruker, 2005 ▶) *T*_min_ = 0.912, *T*_max_ = 0.962
27543 measured reflections
5088 independent reflections
2909 reflections with *I* > 2σ(*I*)
*R*_int_ = 0.044

#### Refinement

*R*[*F* ^2^ > 2σ(*F* ^2^)] = 0.054
*wR*(*F* ^2^) = 0.144
*S* = 1.02
5088 reflections
237 parameters
H atoms treated by a mixture of independent and constrained refinement
Δρ_max_ = 0.23 e Å^−3^
Δρ_min_ = −0.19 e Å^−3^

### 

Data collection: *APEX2* (Bruker, 2005 ▶); cell refinement: *SAINT* (Bruker, 2005 ▶); data reduction: *SAINT*; program(s) used to solve structure: *SHELXTL* (Sheldrick, 2008 ▶); program(s) used to refine structure: *SHELXTL*; molecular graphics: *SHELXTL*; software used to prepare material for publication: *SHELXTL* and *PLATON* (Spek, 2009 ▶).

## Supplementary Material

Crystal structure: contains datablocks global, I. DOI: 10.1107/S1600536809050090/lh2959sup1.cif

Structure factors: contains datablocks I. DOI: 10.1107/S1600536809050090/lh2959Isup2.hkl

Additional supplementary materials:  crystallographic information; 3D view; checkCIF report

## Figures and Tables

**Table 1:** Hydrogen-bond geometry (Å, °)

*D*—H⋯*A*	*D*—H	H⋯*A*	*D*⋯*A*	*D*—H⋯*A*
N1—H1*N*1⋯O1^i^	0.93 (2)	1.85 (2)	2.774 (2)	178 (2)
N4—H1*N*4⋯O2^ii^	0.88 (2)	1.85 (2)	2.736 (2)	177.2 (14)
C10—H10*A*⋯S1	0.93	2.43	3.203 (2)	140
C16—H16*A*⋯O2^iii^	0.96	2.48	3.368 (4)	153

Symmetry codes: (i) 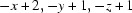; (ii) 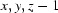; (iii) 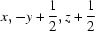.

**Fig. 1.**
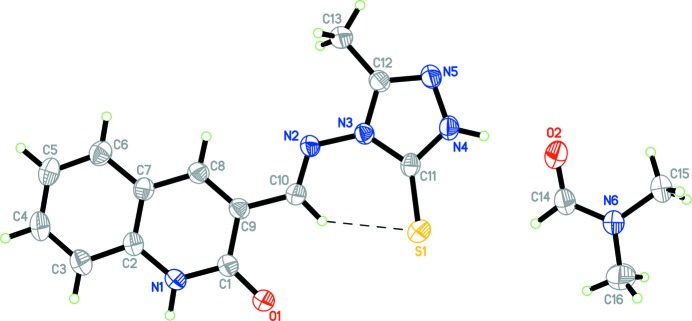
The molecular structure of the title compound, showing 30% probability displacement ellipsoids for non-H atoms and the atom-numbering scheme. An intramolecular hydrogen bond is shown as dashed line.

**Fig. 2.**
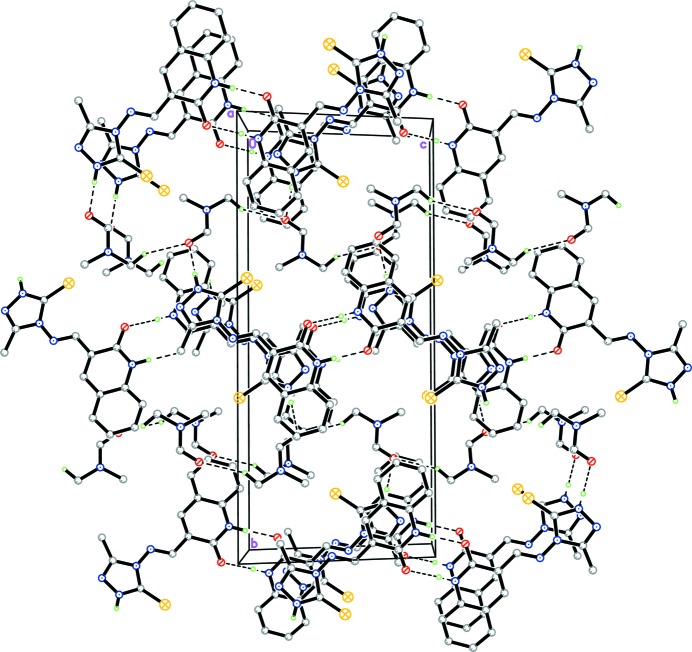
Part of the crystal structure of the title compound, viewed along the a axis, showing dimers being linked into three-dimensional network. H atoms not involved in intermolecular interactions (dashed lines) have been omitted for clarity.

## supplementary crystallographic information

### Comment

A degree of respectability has been bestowed upon 1,2,4-triazole derivatives due to their anti-bacterial, anti-fungal (Turan-Zitouni *et al.*, 2005), anti-tubercular (Walczak *et al.*, 2004), anti-cancer (Holla *et al.*, 2003), anti-tumor (Al-Soud *et al.*, 2003), anti-convulsant (Almasirad *et al.*, 2004), anti-inflammatory and analgesic (Amir & Shikha, 2004) properties. Similarly quinoline and its derivatives have been reported to be associated with interesting pharmacological properties (Janardhana *et al.*, 2008; Kalluraya & Sreenivasa, 1998). They are found in numerous commercial products, including pharmaceuticals, frangrances and dyes. Schiff base derivatives of 1,2,4-triazol-5-ones are also found to possess anti-tumor (Demirbas *et al.*, 2004) and anti-inflammatory (Sujith *et al.*, 2009) activities. These observations prompted us to synthesize the title compound and to characterize it by single crystal XRD study.

The asymmetric unit of the title compound (Fig. 1) comprises of a 4-[(2-hydroxyquinolin-3-yl)methyleneamino]-3-methyl-1*H*-1,2,4-triazole- 5(4*H*)-thione molecule and a *N*,*N*-dimethylformamide solvent molecule. In the main molecule, exists in a *trans* configuration with respect to the acyclic C10═N2 bond. An intramolecular C10—H10A···S1 hydrogen bond (Table 1) generates a six-membered ring, producing an *S*(6) ring motif (Fig. 1, Berstein *et al.*, 1995). A proton is transferred from atom O1 of the hydroxy group to atom N1. Comparing with the unprotonated structure (Dufresne *et al.*, 2008), protonation of atom N1 has widened the C1—N1—C2 angle from 117.25 (14) to 124.83 (18)°. The 1,2,4-triazole ring and quinoline ring system are essentially planar, with maximum deviations of 0.001 (2) and 0.013 (2) Å, respectively, for atoms N3 and C6. These two ring systems are slightly inclined to one another at a dihedral angle of 5.86 (9)°. The bond lengths and angles are comparable to those related 1,2,4-triazole (Fun *et al.*, 2009) and quinoline (Song *et al.*, 2008) structures.

In the crystal structure (Fig. 2), the protonated N1 atom act as hydrogen bond donor to the O1 atom of an inversion-related molecule, producing an *R*_2_^2^(8) hydrogen-bonded dimer through N1—H1N1···O1^i^ hydrogen bond (see Table 1 for symmetry codes). The *N*,*N*-dimethylformamide solvent molecules further link these dimers *via* intermolecular N4—H1N4···O2^ii^ and C16—H16A···O2^iii^ hydrogen bonds (Table 1), establishing connections within these dimers and thus creating a three dimensional network. The crystal structure is consolidated by two different weak intermolecular π–π interactions involving the 1,2,4-triazole (*Cg*1) and C1/N1/C2/C7-C9 pyridine (*Cg*2) rings [*Cg*1···*Cg*2^iv^ = 3.6593 (12) and *Cg*1···*Cg*2^v^ = 3.6892 (12) Å, respectively; (iv) 2-x, 1-y, -z and (v) 1-x, 1-y, -z].

### Experimental

The title compound was obtained by refluxing 3-methyl-4-amino-1,2,4-triazole-5-thione (0.01 mol) and 2-hydroxy-3-formyl-quinoline (0.01 mol) in ethanol (30 ml) with the addition of three drops of concentrated sulphuric acid for 3 h. The solid product obtained was collected by filtration, washed with ethanol and dried. It was then recrystallized using ethanol. Single crystals suitable for X-ray analysis were obtained from a solution of the title compound in a mixture of ethanol and DMF by slow evaporation.

### Refinement

Atoms H1N1 and H1N4 were located from difference Fourier map and allowed to refine freely. All other hydrogen atoms were placed in calculated positions, with C—H = 0.93 – 0.96 Å, and refined using a riding model, with *U*_iso_ = 1.2 or 1.5 *U*_eq_(C). A rotating group model was used for the methyl groups. The reflection (020) was omitted as the intensity was affected by the beam backstop. The highest residual electron density peak and the deepest hole are located at 1.02 and 0.42 Å, respectively, from the sulphur atom.

### Crystal data

**Table:**

C_13_H_11_N_5_OS·C_3_H_7_NO	*F*(000) = 752
*M**_r_* = 358.42	*D*_x_ = 1.359 Mg m^−^^3^
Monoclinic, *P*2_1_/*c*	Mo *K*α radiation, λ = 0.71073 Å
Hall symbol: -P 2ybc	Cell parameters from 5142 reflections
*a* = 7.2374 (1) Å	θ = 2.6–24.1°
*b* = 23.4970 (4) Å	µ = 0.21 mm^−^^1^
*c* = 10.8214 (2) Å	*T* = 296 K
β = 107.820 (1)°	Block, orange
*V* = 1751.97 (5) Å^3^	0.45 × 0.27 × 0.19 mm
*Z* = 4	

### Data collection

**Table:**

Bruker SMART APEXII CCD area-detector diffractometer	5088 independent reflections
Radiation source: fine-focus sealed tube	2909 reflections with *I* > 2σ(*I*)
graphite	*R*_int_ = 0.044
φ and ω scans	θ_max_ = 30.0°, θ_min_ = 2.2°
Absorption correction: multi-scan (*SADABS*; Bruker, 2005)	*h* = −10→10
*T*_min_ = 0.912, *T*_max_ = 0.962	*k* = −32→33
27543 measured reflections	*l* = −14→15

### Refinement

**Table:**

Refinement on *F*^2^	Primary atom site location: structure-invariant direct methods
Least-squares matrix: full	Secondary atom site location: difference Fourier map
*R*[*F*^2^ > 2σ(*F*^2^)] = 0.054	Hydrogen site location: inferred from neighbouring sites
*wR*(*F*^2^) = 0.144	H atoms treated by a mixture of independent and constrained refinement
*S* = 1.02	*w* = 1/[σ^2^(*F*_o_^2^) + (0.0578*P*)^2^ + 0.3261*P*] where *P* = (*F*_o_^2^ + 2*F*_c_^2^)/3
5088 reflections	(Δ/σ)_max_ < 0.001
237 parameters	Δρ_max_ = 0.23 e Å^−^^3^
0 restraints	Δρ_min_ = −0.19 e Å^−^^3^

### Special details

**Table:**

Geometry. All esds (except the esd in the dihedral angle between two l.s. planes) are estimated using the full covariance matrix. The cell esds are taken into account individually in the estimation of esds in distances, angles and torsion angles; correlations between esds in cell parameters are only used when they are defined by crystal symmetry. An approximate (isotropic) treatment of cell esds is used for estimating esds involving l.s. planes.
Refinement. Refinement of F^2^ against ALL reflections. The weighted R-factor wR and goodness of fit S are based on F^2^, conventional R-factors R are based on F, with F set to zero for negative F^2^. The threshold expression of F^2^ > 2sigma(F^2^) is used only for calculating R-factors(gt) etc. and is not relevant to the choice of reflections for refinement. R-factors based on F^2^ are statistically about twice as large as those based on F, and R- factors based on ALL data will be even larger.

### Fractional atomic coordinates and isotropic or equivalent isotropic displacement parameters (Å^2^)

**Table:**

	*x*	*y*	*z*	*U*_iso_*/*U*_eq_	
S1	0.62071 (9)	0.36346 (2)	0.02621 (6)	0.0699 (2)	
O1	0.9054 (2)	0.46985 (6)	0.34539 (13)	0.0699 (4)	
N1	1.0341 (2)	0.55794 (7)	0.39613 (17)	0.0555 (4)	
N2	0.7262 (2)	0.50131 (6)	−0.03966 (15)	0.0485 (4)	
N3	0.6254 (2)	0.45953 (6)	−0.12480 (14)	0.0452 (3)	
N4	0.4855 (2)	0.38697 (7)	−0.22893 (17)	0.0554 (4)	
N5	0.4700 (2)	0.42818 (7)	−0.32108 (16)	0.0586 (4)	
C1	0.9416 (3)	0.51769 (8)	0.30855 (19)	0.0534 (5)	
C2	1.0822 (3)	0.61175 (8)	0.36456 (19)	0.0510 (4)	
C3	1.1770 (3)	0.65002 (9)	0.4618 (2)	0.0641 (5)	
H3A	1.2079	0.6397	0.5488	0.077*	
C4	1.2241 (3)	0.70310 (9)	0.4275 (2)	0.0698 (6)	
H4A	1.2854	0.7290	0.4920	0.084*	
C5	1.1817 (3)	0.71879 (9)	0.2983 (3)	0.0680 (6)	
H5A	1.2162	0.7547	0.2769	0.082*	
C6	1.0893 (3)	0.68142 (8)	0.2023 (2)	0.0617 (5)	
H6A	1.0612	0.6922	0.1157	0.074*	
C7	1.0366 (3)	0.62699 (8)	0.23332 (19)	0.0497 (4)	
C8	0.9380 (3)	0.58604 (8)	0.13906 (19)	0.0505 (4)	
H8A	0.9049	0.5956	0.0516	0.061*	
C9	0.8906 (3)	0.53351 (7)	0.17255 (17)	0.0472 (4)	
C10	0.7858 (3)	0.49056 (8)	0.07984 (19)	0.0526 (5)	
H10A	0.7630	0.4549	0.1095	0.063*	
C11	0.5783 (2)	0.40326 (7)	−0.10719 (19)	0.0485 (4)	
C12	0.5562 (3)	0.47203 (8)	−0.25541 (19)	0.0516 (4)	
C13	0.5759 (3)	0.52850 (9)	−0.3093 (2)	0.0685 (6)	
H13A	0.5120	0.5284	−0.4012	0.103*	
H13B	0.7109	0.5372	−0.2928	0.103*	
H13C	0.5175	0.5567	−0.2690	0.103*	
O2	0.3433 (2)	0.27901 (6)	0.71425 (18)	0.0801 (5)	
N6	0.2576 (2)	0.19916 (7)	0.79911 (17)	0.0606 (4)	
C14	0.3236 (3)	0.25191 (9)	0.8062 (2)	0.0645 (6)	
H14A	0.3576	0.2697	0.8867	0.077*	
C15	0.1949 (4)	0.17088 (10)	0.6743 (2)	0.0841 (7)	
H15A	0.1598	0.1988	0.6065	0.126*	
H15B	0.0847	0.1473	0.6696	0.126*	
H15C	0.2987	0.1478	0.6643	0.126*	
C16	0.2369 (5)	0.16951 (13)	0.9100 (3)	0.1056 (10)	
H16A	0.2538	0.1957	0.9806	0.158*	
H16B	0.3333	0.1401	0.9350	0.158*	
H16C	0.1099	0.1528	0.8886	0.158*	
H1N1	1.054 (3)	0.5478 (9)	0.482 (2)	0.063 (6)*	
H1N4	0.436 (3)	0.3526 (10)	−0.249 (2)	0.070 (7)*	

### Atomic displacement parameters (Å^2^)

**Table:**

	*U*^11^	*U*^22^	*U*^33^	*U*^12^	*U*^13^	*U*^23^
S1	0.0962 (4)	0.0476 (3)	0.0632 (4)	−0.0040 (3)	0.0204 (3)	0.0050 (2)
O1	0.0986 (11)	0.0555 (8)	0.0482 (8)	−0.0161 (7)	0.0116 (8)	0.0012 (7)
N1	0.0672 (10)	0.0548 (10)	0.0410 (9)	−0.0070 (8)	0.0115 (8)	−0.0036 (8)
N2	0.0560 (9)	0.0419 (8)	0.0454 (9)	−0.0008 (6)	0.0122 (7)	−0.0048 (7)
N3	0.0482 (8)	0.0424 (8)	0.0445 (9)	0.0000 (6)	0.0133 (7)	−0.0046 (6)
N4	0.0597 (10)	0.0435 (9)	0.0593 (11)	−0.0032 (7)	0.0128 (8)	−0.0085 (8)
N5	0.0678 (10)	0.0497 (9)	0.0525 (10)	−0.0019 (7)	0.0098 (8)	−0.0063 (8)
C1	0.0603 (11)	0.0495 (11)	0.0479 (11)	−0.0028 (8)	0.0131 (9)	−0.0040 (9)
C2	0.0468 (10)	0.0499 (10)	0.0550 (12)	−0.0008 (8)	0.0137 (8)	−0.0072 (9)
C3	0.0641 (12)	0.0643 (13)	0.0594 (13)	−0.0034 (10)	0.0124 (10)	−0.0133 (10)
C4	0.0643 (13)	0.0584 (13)	0.0811 (17)	−0.0099 (10)	0.0139 (12)	−0.0210 (12)
C5	0.0706 (13)	0.0472 (11)	0.0881 (18)	−0.0082 (10)	0.0272 (12)	−0.0089 (11)
C6	0.0677 (12)	0.0519 (11)	0.0688 (14)	−0.0018 (9)	0.0258 (11)	−0.0025 (10)
C7	0.0486 (10)	0.0457 (10)	0.0561 (12)	0.0001 (7)	0.0181 (9)	−0.0043 (8)
C8	0.0568 (11)	0.0498 (10)	0.0458 (11)	0.0006 (8)	0.0169 (9)	−0.0025 (9)
C9	0.0509 (10)	0.0466 (10)	0.0433 (10)	0.0013 (8)	0.0135 (8)	−0.0030 (8)
C10	0.0622 (11)	0.0441 (10)	0.0509 (12)	−0.0029 (8)	0.0166 (9)	−0.0017 (8)
C11	0.0482 (9)	0.0399 (9)	0.0577 (12)	0.0024 (7)	0.0166 (9)	−0.0059 (8)
C12	0.0556 (10)	0.0506 (10)	0.0456 (11)	0.0018 (8)	0.0109 (8)	−0.0032 (9)
C13	0.0890 (15)	0.0562 (12)	0.0549 (13)	−0.0040 (11)	0.0137 (11)	0.0037 (10)
O2	0.0986 (12)	0.0576 (9)	0.0853 (12)	−0.0178 (8)	0.0299 (10)	−0.0067 (9)
N6	0.0706 (11)	0.0506 (9)	0.0597 (11)	−0.0023 (8)	0.0185 (9)	−0.0055 (8)
C14	0.0645 (13)	0.0587 (13)	0.0658 (15)	−0.0008 (10)	0.0131 (11)	−0.0148 (11)
C15	0.114 (2)	0.0615 (14)	0.0710 (16)	−0.0128 (13)	0.0200 (14)	−0.0153 (12)
C16	0.153 (3)	0.094 (2)	0.082 (2)	−0.0127 (19)	0.0536 (19)	0.0069 (16)

### Geometric parameters (Å, °)

**Table:**

S1—C11	1.668 (2)	C6—C7	1.405 (3)
O1—C1	1.247 (2)	C6—H6A	0.9300
N1—C1	1.361 (2)	C7—C8	1.425 (2)
N1—C2	1.382 (2)	C8—C9	1.359 (2)
N1—H1N1	0.93 (2)	C8—H8A	0.9300
N2—C10	1.257 (2)	C9—C10	1.461 (2)
N2—N3	1.3903 (19)	C10—H10A	0.9300
N3—C12	1.379 (2)	C12—C13	1.474 (3)
N3—C11	1.393 (2)	C13—H13A	0.9600
N4—C11	1.338 (2)	C13—H13B	0.9600
N4—N5	1.370 (2)	C13—H13C	0.9600
N4—H1N4	0.88 (2)	O2—C14	1.225 (3)
N5—C12	1.298 (2)	N6—C14	1.322 (3)
C1—C9	1.452 (3)	N6—C16	1.434 (3)
C2—C3	1.395 (3)	N6—C15	1.448 (3)
C2—C7	1.403 (3)	C14—H14A	0.9300
C3—C4	1.374 (3)	C15—H15A	0.9600
C3—H3A	0.9300	C15—H15B	0.9600
C4—C5	1.386 (3)	C15—H15C	0.9600
C4—H4A	0.9300	C16—H16A	0.9600
C5—C6	1.369 (3)	C16—H16B	0.9600
C5—H5A	0.9300	C16—H16C	0.9600
			
C1—N1—C2	124.83 (18)	C8—C9—C10	124.36 (17)
C1—N1—H1N1	114.3 (13)	C1—C9—C10	115.88 (16)
C2—N1—H1N1	120.6 (13)	N2—C10—C9	120.68 (17)
C10—N2—N3	119.03 (16)	N2—C10—H10A	119.7
C12—N3—N2	118.77 (15)	C9—C10—H10A	119.7
C12—N3—C11	108.36 (15)	N4—C11—N3	101.93 (16)
N2—N3—C11	132.85 (15)	N4—C11—S1	126.57 (14)
C11—N4—N5	114.79 (16)	N3—C11—S1	131.50 (14)
C11—N4—H1N4	123.0 (15)	N5—C12—N3	110.81 (17)
N5—N4—H1N4	122.2 (15)	N5—C12—C13	125.92 (18)
C12—N5—N4	104.12 (16)	N3—C12—C13	123.26 (17)
O1—C1—N1	120.69 (18)	C12—C13—H13A	109.5
O1—C1—C9	122.81 (17)	C12—C13—H13B	109.5
N1—C1—C9	116.50 (17)	H13A—C13—H13B	109.5
N1—C2—C3	120.42 (19)	C12—C13—H13C	109.5
N1—C2—C7	119.00 (17)	H13A—C13—H13C	109.5
C3—C2—C7	120.58 (18)	H13B—C13—H13C	109.5
C4—C3—C2	119.1 (2)	C14—N6—C16	122.4 (2)
C4—C3—H3A	120.4	C14—N6—C15	119.25 (19)
C2—C3—H3A	120.4	C16—N6—C15	118.30 (19)
C3—C4—C5	121.2 (2)	O2—C14—N6	124.8 (2)
C3—C4—H4A	119.4	O2—C14—H14A	117.6
C5—C4—H4A	119.4	N6—C14—H14A	117.6
C6—C5—C4	120.1 (2)	N6—C15—H15A	109.5
C6—C5—H5A	120.0	N6—C15—H15B	109.5
C4—C5—H5A	120.0	H15A—C15—H15B	109.5
C5—C6—C7	120.5 (2)	N6—C15—H15C	109.5
C5—C6—H6A	119.7	H15A—C15—H15C	109.5
C7—C6—H6A	119.7	H15B—C15—H15C	109.5
C2—C7—C6	118.54 (18)	N6—C16—H16A	109.5
C2—C7—C8	117.62 (17)	N6—C16—H16B	109.5
C6—C7—C8	123.84 (19)	H16A—C16—H16B	109.5
C9—C8—C7	122.29 (18)	N6—C16—H16C	109.5
C9—C8—H8A	118.9	H16A—C16—H16C	109.5
C7—C8—H8A	118.9	H16B—C16—H16C	109.5
C8—C9—C1	119.75 (17)		
			
C10—N2—N3—C12	178.43 (17)	O1—C1—C9—C8	−178.68 (18)
C10—N2—N3—C11	−3.3 (3)	N1—C1—C9—C8	0.8 (3)
C11—N4—N5—C12	0.1 (2)	O1—C1—C9—C10	2.5 (3)
C2—N1—C1—O1	179.27 (18)	N1—C1—C9—C10	−178.08 (16)
C2—N1—C1—C9	−0.2 (3)	N3—N2—C10—C9	−179.39 (15)
C1—N1—C2—C3	179.90 (18)	C8—C9—C10—N2	−2.6 (3)
C1—N1—C2—C7	−0.9 (3)	C1—C9—C10—N2	176.18 (17)
N1—C2—C3—C4	179.58 (19)	N5—N4—C11—N3	−0.2 (2)
C7—C2—C3—C4	0.4 (3)	N5—N4—C11—S1	179.90 (13)
C2—C3—C4—C5	−1.0 (3)	C12—N3—C11—N4	0.18 (18)
C3—C4—C5—C6	0.8 (3)	N2—N3—C11—N4	−178.25 (16)
C4—C5—C6—C7	0.0 (3)	C12—N3—C11—S1	−179.91 (15)
N1—C2—C7—C6	−178.83 (17)	N2—N3—C11—S1	1.7 (3)
C3—C2—C7—C6	0.3 (3)	N4—N5—C12—N3	0.0 (2)
N1—C2—C7—C8	1.4 (3)	N4—N5—C12—C13	−178.53 (19)
C3—C2—C7—C8	−179.39 (16)	N2—N3—C12—N5	178.56 (15)
C5—C6—C7—C2	−0.5 (3)	C11—N3—C12—N5	−0.1 (2)
C5—C6—C7—C8	179.16 (18)	N2—N3—C12—C13	−2.9 (3)
C2—C7—C8—C9	−0.9 (3)	C11—N3—C12—C13	178.46 (18)
C6—C7—C8—C9	179.39 (18)	C16—N6—C14—O2	−179.8 (2)
C7—C8—C9—C1	−0.2 (3)	C15—N6—C14—O2	−2.8 (3)
C7—C8—C9—C10	178.53 (16)		

### Hydrogen-bond geometry (Å, °)

**Table:**

*D*—H···*A*	*D*—H	H···*A*	*D*···*A*	*D*—H···*A*
N1—H1N1···O1^i^	0.93 (2)	1.85 (2)	2.774 (2)	178 (2)
N4—H1N4···O2^ii^	0.88 (2)	1.85 (2)	2.736 (2)	177.2 (14)
C10—H10A···S1	0.93	2.43	3.203 (2)	140
C16—H16A···O2^iii^	0.96	2.48	3.368 (4)	153

Symmetry codes: (i) −*x*+2, −*y*+1, −*z*+1; (ii) *x*, *y*, *z*−1; (iii) *x*, −*y*+1/2, *z*+1/2.
